# Length Bias Correction in Gene Ontology Enrichment Analysis Using Logistic Regression

**DOI:** 10.1371/journal.pone.0046128

**Published:** 2012-10-02

**Authors:** Gu Mi, Yanming Di, Sarah Emerson, Jason S. Cumbie, Jeff H. Chang

**Affiliations:** 1 Department of Statistics, Oregon State University, Corvallis, Oregon, United States of America; 2 Molecular and Cellular Biology Program, Oregon State University, Corvallis, Oregon, United States of America; 3 Department of Botany and Plant Pathology, Oregon State University, Corvallis, Oregon, United States of America; 4 Center for Genome Research and Biocomputing, Oregon State University, Corvallis, Oregon, United States of America; Biodiversity Insitute of Ontario - University of Guelph, Canada

## Abstract

When assessing differential gene expression from RNA sequencing data, commonly used statistical tests tend to have greater power to detect differential expression of genes encoding longer transcripts. This phenomenon, called “length bias”, will influence subsequent analyses such as Gene Ontology enrichment analysis. In the presence of length bias, Gene Ontology categories that include longer genes are more likely to be identified as enriched. These categories, however, are not necessarily biologically more relevant. We show that one can effectively adjust for length bias in Gene Ontology analysis by including transcript length as a covariate in a logistic regression model. The logistic regression model makes the statistical issue underlying length bias more transparent: transcript length becomes a confounding factor when it correlates with both the Gene Ontology membership and the significance of the differential expression test. The inclusion of the transcript length as a covariate allows one to investigate the direct correlation between the Gene Ontology membership and the significance of testing differential expression, conditional on the transcript length. We present both real and simulated data examples to show that the logistic regression approach is simple, effective, and flexible.

## Introduction

RNA sequencing (RNA-Seq) has the potential to enable simultaneous measurement of expression for all genes expressed in a cell. Statistical tests [1]–[3] further enable assessment of differential expression (DE) of individual genes under different environmental or experimental conditions. To relate the outcome of the DE analysis to biological functions, a widely-used approach is to examine enriched Gene Ontology (GO) categories based on the terms annotated to the genes identified as DE [4], [5]. GO uses a structured vocabulary to describe functional categories of gene products. Genes annotated with the same GO term form a gene category and share a common biological function. The enrichment of a GO term among DE genes can be used to indicate the association of biological functions to variations in experimental conditions.

**Figure 1:**
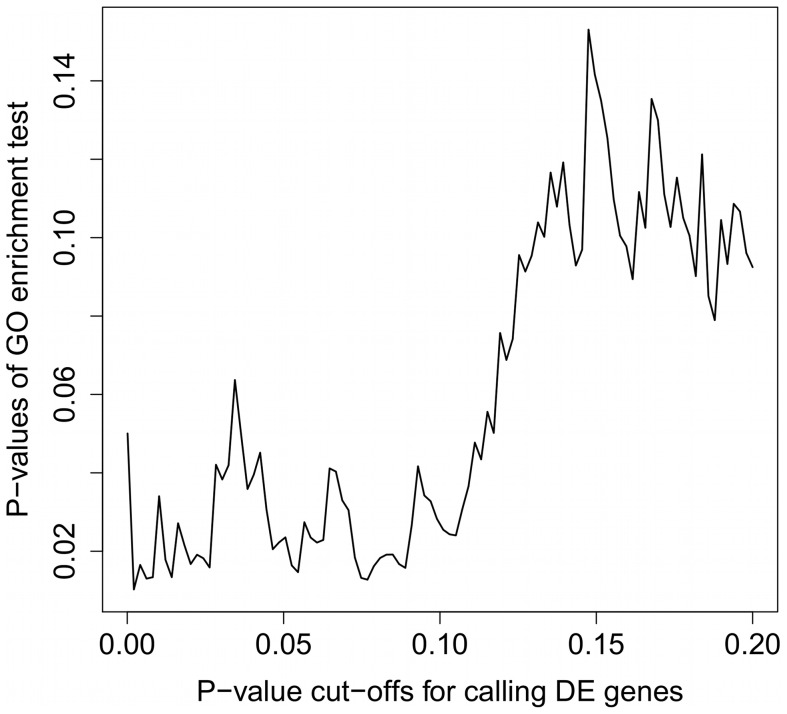
Influence of DE testing 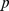-value thresholds on the determination of enriched categories. The 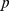-value cut-off for calling DE genes (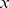-axis) influences the 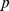-value of subsequent GO enrichment test (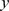-axis). Therefore, subjective decisions on declaring DE genes will make subsequent enrichment results rather unstable.

To quantify the enrichment of a GO term, one can dichotomize results of DE analysis and cross-classify the genes according to whether they are indicated as DE and whether they are annotated to the specific GO term. The level of enrichment can then be assessed using contingency-table-based tests such as the Fisher's exact test or the chi-square test (for a summary, see [6]). Table 1 shows an example of testing the enrichment of the GO term GO:0005575 among DE genes in a prostate cancer dataset (see the Results section for more details). One unappealing feature of the contingency-table-based approach is that the numbers of DE and non-DE genes and, in turn, the GO enrichment test result depend on the 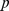-value cut-off for declaring a gene as DE. In Table 1, genes with DE test 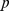-values less than 0.05 are declared as DE. Figure 1 shows that Fisher's exact test 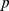-values vary with DE test 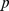-value cut-offs.

**Table 1:** A typical two-by-two contingency table for testing enrichment of a GO category.

	*D*	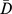	Sum
*C*	1962	9803	11765
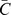	118	709	827
Sum	2080	10512	12592

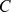
: in category;

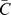
: not in category;

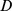
: DE genes;

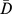
: non-DE genes.

Logistic regression is an alternative GO enrichment analysis approach that does not require dichotomizing DE test results. For each gene 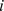, let the binary variable 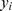 indicate the presence (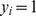) or absence (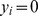) of the gene in the GO category. Denote 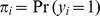, and let 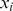 measure the significance of the DE test result (e.g., transformation of the DE test 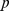-value). The logistic regression [7]

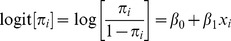
(1)relates the log odds of a gene belonging to a GO category to the significance of DE tests. A significant positive 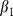 indicates that the odds of a gene belonging to this particular category increase as the significance of DE increases. Sartor *et al.* implemented the logistic regression model in the software LRpath and applied it to enrichment analyses for microarray expression data [8]. The logistic regression approach is more flexible than the contingency table approach. First, it is easy to include covariates in a logistic regression setting to adjust for potential confounding factors–such as gene length. Second, logistic regression allows the use of continuous measures of DE test significance, which conveys more information than dichotomizing DE test results and avoids the nuisance of choosing an arbitrary cut-off for DE test 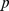-values.

One statistical concern with tests for enriched GO terms, particularly those based on analysis of RNA-Seq datasets, is that transcript length can be a confounding factor if it correlates with both the GO membership and the DE test significance. In regards to the latter, many existing DE tests have greater statistical power to detect DE for genes with more reads mapped to them [1]–[3]. Since genes with longer transcripts will have more reads mapped to them than an equally expressed shorter gene, the statistical *power* of these DE tests will depend on transcript lengths. Oshlack *et al.* refer to the dependence of DE test power on transcript length as length bias [9]. In the presence of length bias, subsequent GO enrichment analysis will have the potential to identify GO categories with a higher proportion of longer genes. These categories are not necessarily biologically more relevant.

Young *et al.* compensated for potential length bias by developing a weighted resampling strategy based on contingency tables [10]. The basic idea is to estimate the DE test power as a smooth function of the transcript length and resample genes with weights inversely proportional to the estimated power of the DE test. For computational efficiency, the resampling method can be approximated by a test based on the Wallenius non-central hypergeometric distribution. If no length bias is present, the Wallenius approximation reduces to the Fisher's exact test, which is based on a central hypergeometric distribution. Their method is implemented in the Bioconductor package goseq. Gao *et al.* proposed a similar method where a different weighting function is used to compute the non-central parameter of the Wallenius distribution [11].

The dependencies between the GO terms should also be considered in the statistical assessment for enrichment of GO categories. GO terms are organized as a directed acyclic graph (DAG). In this DAG, parent terms describe more general functional categories than their child terms [12] and each child term can have multiple parent terms. Because of this relationship, a gene, when annotated with a GO term, is automatically annotated to the term's parent terms. Furthermore, any gene has the potential for being annotated with multiple GO terms. Three distinct categories, *biological process* (BP), *cellular component* (CC) and *molecular function* (MF), describe the most general biological functions each having potentially thousands of annotated genes, while some of the very specialized categories (e.g. *inner membrane complex*) may even have no gene products annotated to them. When a GO term describing a general biological function is identified as enriched among DE genes, all its offspring–GO terms describing more specific functions–tend to be enriched as well. As a result, if we rank the GO terms according to enrichment test 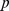-values, we tend to see many specific terms at the top of the list, which may result in potentially misleading interpretation of the data. To address this issue, Alexa *et al.* [13] and Grossmann *et al.* [14] proposed “local” GO enrichment tests that incorporate the parent-child relationship among GO terms. The basic idea is to examine the relative enrichment of a GO term among genes that are offspring of the parents of the GO term being tested. If a GO term is enriched only because its parents are, the local GO enrichment test will not identify it as enriched. The topGO Bioconductor package [15] implements the method proposed in [13], and the Ontologizer2 software [16] implements methods proposed in [14].

In this paper, we describe our development of GOglm, a logistic regression model that effectively adjusts for length bias by including transcript length as a covariate. This inclusion allows one to investigate the direct correlation between the GO membership and the DE test significance conditional on the transcript length. We analyzed two public RNA-Seq datasets and simulated data to show that in comparison to the GOseq approach, the GOglm method for length bias corrections is equally effective, but confers the advantages of being more simple, transparent and flexible. We also show that the flexibility of GOglm allows one to address dependences in the GO terms.

## Results

### Length Bias Correction Using Logistic Regression

We propose to adjust for length bias using logistic regression. The method is simple and effective, allows the use of continuous measures of DE test significance, and is flexible to incorporate the parent-child relationship among GO terms.

Correcting length bias using logistic regression is straightforward. One includes a measure of gene length, 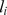, as a covariate in the logistic regression model:
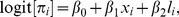
(2)where 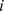 indexes genes, 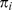 is the probability of a gene belonging to the specified GO category, and 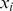 measures the significance of the DE test result. In GOglm, a gene's length is defined as the median length of all its corresponding mature transcripts.

The logistic regression model is easy to interpret and makes the underlying statistical issue more transparent: the fundamental cause of length bias is that the transcript length becomes a confounding factor when it correlates with both the GO membership and the DE test significance. When we include transcript length as a covariate, the coefficient 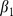 now captures the correlation between the log odds of being in the specified GO category and the DE test significance–conditional on gene lengths. A significant result from the hypothesis test 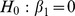 indicates that the GO membership is correlated with the DE test significance even after adjusting for length bias.

The logistic regression method is more flexible than the GOseq approach. The logistic regression can be used in the contingency table setting by letting 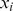 be a binary variable indicating whether gene 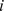 is DE or non-DE. In the data examples below, we will show that the logistic regression method is equally effective in accounting for length bias as the GOseq approach. But more generally, the logistic regression model can use continuous measures of DE significance as explained earlier. In GOglm, one option is to use 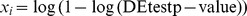 as the continuous measure of DE. The inner log transformation helps us focus attention on the order of magnitude change in 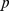-values. The outer log transformation will down weigh influence from extremely small 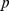-values. There are other ways to construct the significance measure. We discussed earlier that 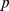-values can be dichotomized, but that will incur loss of information. One can construct the significance measure based on test statistic values (usually, the 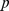-value is a monotone function of the test statistic). Other measures such as log fold change can also be used. We compared the performance of using different measures of DE in the section Simulation Studies.

Logistic regression is flexible to incorporate the parent-child relationship. To test for local enrichment, one fits the logistic regression using a subset of genes. For example, if the set of all genes annotated to the direct parents of the GO term is used to fit the regression model, the results will be similar to the parent-child union approach in Ontologizer2. In the following section on RNA-Seq data examples, we analyze the Arabidopsis data and compare results between GOglm and Ontologizer2.

Most statistical software includes efficient programs for fitting logistic regression models. We implement GOglm in R [17] and fit the logistic regression model using the glm function with quasi-binomial error distribution in order to account for potential over-dispersions. The function glm uses the iteratively weighted least squares method (equivalent to Newton-Raphson algorithm for logistic regression) for estimation of regression coefficients and the Wald test for hypothesis tests of regression coefficients.

When a GO category contains very few (e.g., fewer than 5) annotated genes, the enrichment test 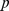-value can be volatile and the statistical evidence can be unreliable. In GOglm, users have the option to exclude GO categories with too few genes when ranking the enrichment test results. Other GO analysis software such as topGO [15] and LRpath [8] also allow users to filter out GO categories with small sizes.

### RNA-Seq Data Examples

We present results to demonstrate the effectiveness and flexibility of the GOglm logistic regression method for GO enrichment analysis. First, we present results from GO enrichment analysis of the prostate cancer data [18]. Young *et al.* used this dataset to validate their GOseq method [10]. We compare the performance of GOglm and GOseq and demonstrate that the GOglm method effectively accounts for length bias. Second, we use GOglm to perform local enrichment test on an Arabidopsis dataset [3] by examining a GO term's relative enrichment in the context of its direct parent(s). The results are compared to results derived from Ontologizer2 [14].

#### Prostate cancer data example

The prostate cancer data of [18] contain RNA-Seq reads that aligned to 49506 genes from three untreated and four treated cancer samples. It has been demonstrated that the GOseq method is effective in correcting length bias in this dataset [10]. We will use this dataset to compare the performance of GOglm and GOseq.

We used edgeR [1] with a common dispersion estimate to obtain DE test 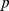-values. The GOseq method was designed for contingency tables, so for the purpose of comparison, the 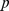-values were dichotomized–a gene was called DE if the FDR adjusted 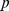-value (i.e. 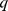-value) was less than 0.05. The same DE results were then used by GOglm and GOseq for GO enrichment analyses.

As discussed in the Introduction section, length bias becomes a confounding factor when it correlates with both the response (GO category membership) and the predictor variable (DE test significance). To illustrate the prevalence of the correlation between GO category and gene length, we considered 4249 categories having at least 10 annotated genes and asked what proportion of these categories showed significant correlation with gene length. For each GO category, we tested the correlation between category membership and gene length by comparing lengths of genes (on log scale) within and out of the category using Welch's two-sample 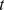-test. We found the proportion to be 44.7%, indicating that significant correlation between gene length and GO category membership is prevalent among GO categories.

**Figure 2:**
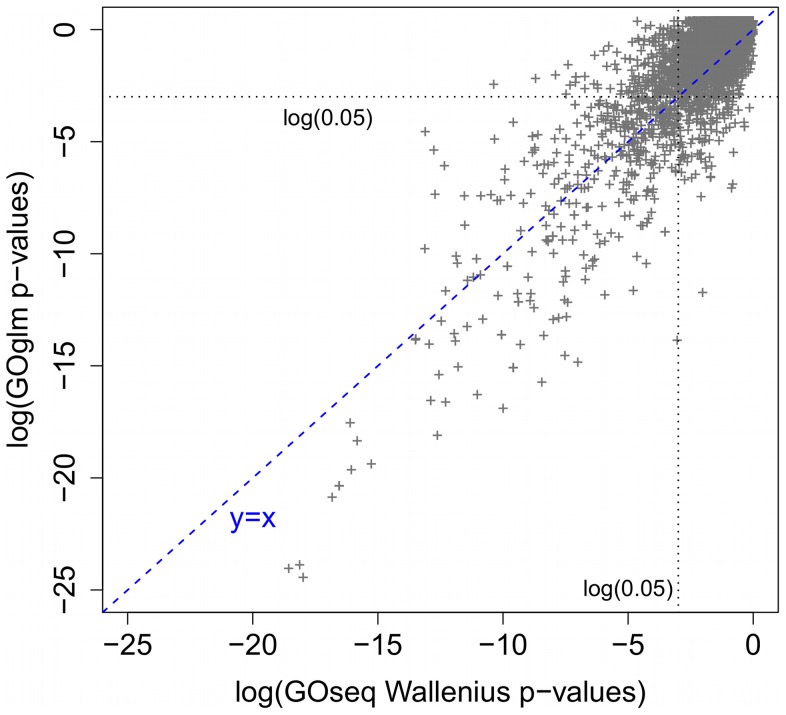
Comparison of 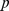-values (on log scale) between GOseq Wallenius and GOglm. Among 3966 GO terms in the prostate cancer dataset, GOseq Wallenius and GOglm detected 492 and 486 enriched categories, respectively. Each plus sign denotes one category.

In Figure 2, we compare GO enrichment test 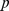-values from the logistic regression method in GOglm and from the Wallenius method in GOseq. There is strong correlation (0.854) between the two sets of 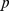-values, especially for small 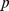-values. In Table 2, we list the most enriched GO terms identified by GOglm, and all of them also rank highly on GOseq's top list.

**Table 2:** Top 10 enriched categories of the prostate cancer dataset as ranked by GOglm.

Accession	Term	Onto^1^	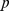 -value	GOglm^2^	GOseq^3^	Leng^4^	Anno^5^
GO:0005737	Cytoplasm	CC	2.30e-13	1	3	2972	6731
GO:0007049	cell cycle	BP	9.19e-12	2	2	3260	1044
GO:0000278	mitotic cell cycle	BP	1.92e-11	3	1	3288	607
GO:0022402	cell cycle process	BP	1.12e-10	4	4	3289	792
GO:0044444	cytoplasmic part	CC	1.41e-10	5	6	2882	4872
GO:0022403	cell cycle phase	BP	2.16e-9	6	8	3226	653
GO:0000087	M phase of mitotic cell cycle	BP	3.59e-9	7	9	3506	303
GO:0000280	nuclear division	BP	5.68e-9	8	12	3555	295
GO:0007067	mitosis	BP	5.68e-9	9	13	3555	295
GO:0048285	organelle fission	BP	8.40e-9	10	16	3516	306

1 BP, biological process; CC, cellular component.

2 GOglm: category ranks by GOglm.

3 GOseq: category ranks by GOseq Wallenius.

4 Leng: median gene length (in base pair) within a category.

5 Anno: number of annotated genes within a category.

**Figure 3:**
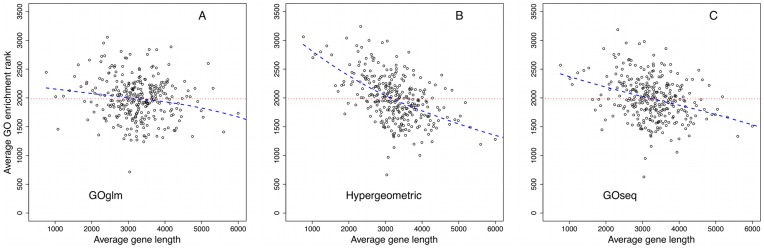
The effect of length bias corrections. GO categories are divided into 300 GO groups based on the average gene length in each category. In each plot, the 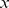-axis represents the average gene length and the 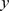-axis represents the average GO enrichment rank in each of the 300 GO groups. The Fisher's exact test (panel B) did not correct for length bias and the enrichment analysis based on this test tended to favor GO categories with longer average lengths. This is reflected as an obvious downward trend in panel B. The downward trend is less pronounced in panels A and C, where GOglm (panel A) and GOseq Wallenius (panel C) were used to adjust for length bias. A horizontal line has been added to each plot to facilitate visual comparison.

Figure 3 demonstrates the effect of length bias corrections. We first ranked the GO categories according to one of the GO enrichment tests: GOglm, GOseq, or the Fisher's exact test. We then divided GO categories into 300 GO groups according to the average gene length in each category and computed the average GO enrichment rank in each group. In Figure 3, we plot the average GO enrichment ranks against the average gene lengths in the 300 GO groups. Panel B shows that the ranks based on the Fisher's exact test–which was not adjusted for length bias–tend to be more biased towards GO categories with greater average gene lengths. This trend is less pronounced in panels A and C, where data were analyzed using GOglm and GOseq, respectively.

#### Arabidopsis data example

The Arabidopsis data contain RNA-Seq reads that aligned to more than 25000 genes from two groups of Arabidopsis samples of size three each. The two groups were derived from plants inoculated with Δ*hrcC* of *Pseudomonas syringae* pv tomato DC3000 or 10 mM MgCl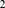 (mock). Di *et al.* performed DE test on this dataset using the NBP negative binomial model [3]. Cumbie *et al.* performed local enrichment analysis on this data using the GORich tool of GENE-Counter [19]. The dataset used in this paper comes directly from [3], which is a subset of the data described in [19].

Here we used the logistic regression in GOglm to perform the local enrichment analysis and compared the results to those derived from Ontologizer2. We focused on up-regulated genes only. Using the R package NBPSeq [20], we performed one-sided DE test to detect up-regulated genes. The logistic regression method in GOglm took continuous measures of DE based on the DE test 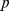-values. In Ontologizer2, DE genes were determined by two criteria: the log fold-change greater than 0 (up-regulated) and the DE testing 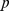-value less than 0.05. In the local enrichment tests, we tested the relative enrichment of a GO term relative to all genes that were offspring of the direct parents of the GO term being tested, which corresponded to the parent-child union (PCU) option in Ontologizer2.

**Figure 4:**
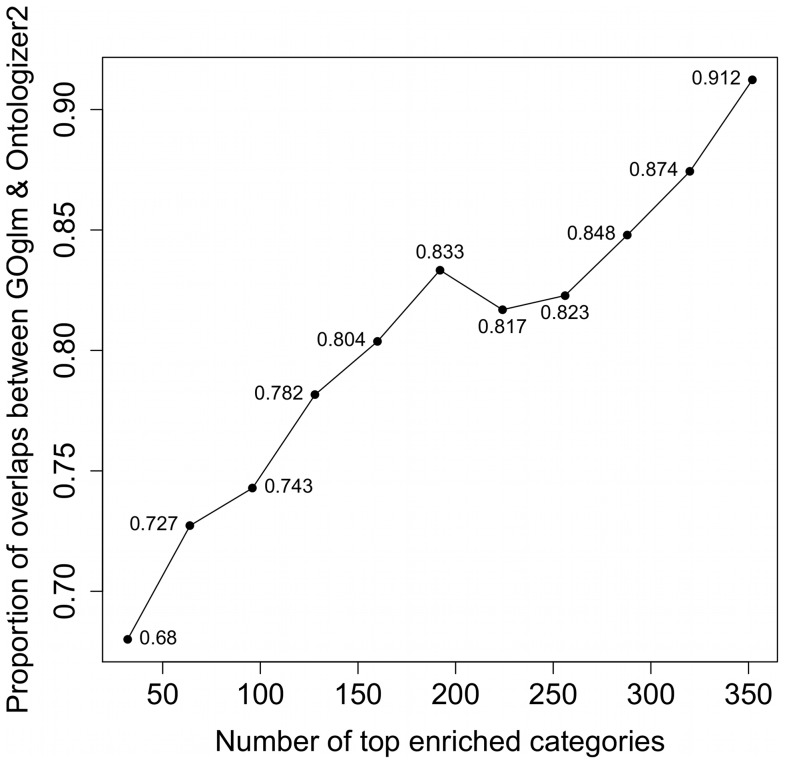
Proportion of overlapping categories by GOglm and Ontologizer2 (PCU). The proportion of overlapping categories (*y*-axis) when the same number (*x*-axis) of top-ranked categories are selected using GOglm and Ontologizer2 (PCU). As more enriched categories were included, there were more overlaps (enriched categories in common) between the two approaches as seen by the increasing trend and the percentages.

From a total of 3851 categories, GOglm and Ontologizer2 detected 358 and 483 enriched categories respectively (using a 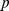-value cut-off of 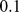 in the enrichment tests). Among the 358 categories identified by GOglm, 201 (56.1%) categories were also declared as enriched by Ontologizer2. The two methods also show high consistency if we focus on the rankings (instead of 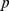-values) of the GO categories. Figure 4 shows the proportion of overlapping categories when the same number of top-ranked categories are selected using each method. Table 3 lists some of the most relevant categories that were declared as enriched by both GOglm and Ontologizer2. Categories such as plant defense, differential expression in response to pathogens, wounding, and/or stresses, signal perception, transduction, secretion or modification of plant cell wall were expected. For the complete ranking lists, see the Supporting Information (Table S1 and Table S2).

**Table 3:** Partial list of enriched categories identified by GOglm in the Arabidopsis dataset.

Accession	Term	Onto^1^	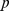 -value	GOglm^2^	Ontgz^3^	Leng^4^	Anno^5^
GO:0050896	response to stimulus	BP	1.20e-19	4	1	1791	4062
GO:0009607	response to biotic stimulus	BP	9.66e-15	6	4	1809	643
GO:0009611	response to wounding	BP	9.66e-13	11	6	1801	164
GO:0045730	respiratory burst	BP	1.78e-7	28	93	1833	6
GO:0009753	response to jasmonic acid stimulus	BP	1.65e-6	37	18	1604	169
GO:0005886	plasma membrane	CC	3.28e-6	41	28	1996	1763
GO:0006952	defense response	BP	5.90e-5	58	39	1940	763
GO:0052482	defense response by cellwall thickening	BP	3.44e-4	69	119	2537	15
GO:0004568	chitinase activity	MF	6.50e-4	78	45	1104	17
GO:0009867	jasmonic acid mediated signaling pathway	BP	1.21e-3	90	150	1711	44

Top 358 and top 483 categories are declared as enriched by GOglm and Ontologizer2 (PCU), respectively.

1 BP, biological process; CC, cellular component; MF, molecular function.

2 GOglm: category ranks by GOglm's local enrichment test.

3 Ontgz: category ranks by Ontologizer2 (PCU).

4 Leng: median gene length (in base pair) within a category.

5 Anno: number of annotated genes within a category.

### Simulation Studies

#### Simulation I

We developed a simulated dataset to further clarify the cause of length bias and demonstrate the effectiveness of our method (GOglm) in correcting the length bias. This dataset consisted of 10426 genes that were binned into 40 non-overlapping categories of different sizes. These 40 categories were simulated such that they varied in the average length of genes, but none of the categories was significantly enriched with DE genes. (See Materials and Methods for further details on the simulation setup.).

To demonstrate the efficacy of our method, we analyzed this simulated dataset using three different methods: 1) a simple logistic regression without length bias corrections (see equation (1)); 2) the GOglm method: logistic regression using log gene length as one additional covariate (see equation (2)); 3) the GOseq method.

**Figure 5:**
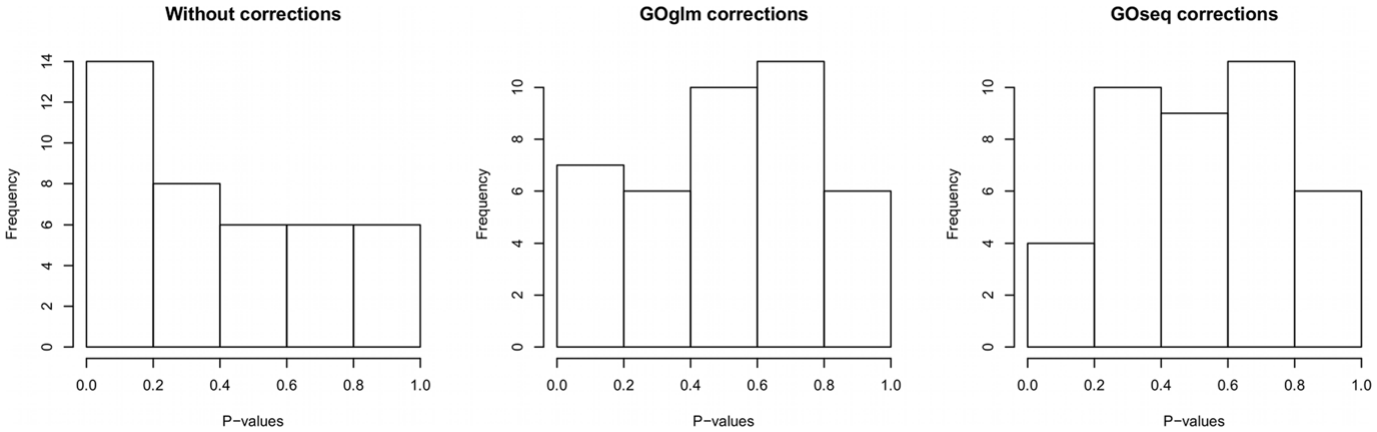
Histograms of enrichment test 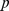-values from three enrichment analysis methods: logistic regression without length bias corrections, GOglm, and GOseq. The left panel (no length bias corrections) shows a more than expected proportion of small 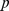-values (false positives). The GOglm (middle panel) and the GOseq (right panel) both gave correct 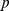-value distributions expected under the simulation setting.

**Figure 6:**
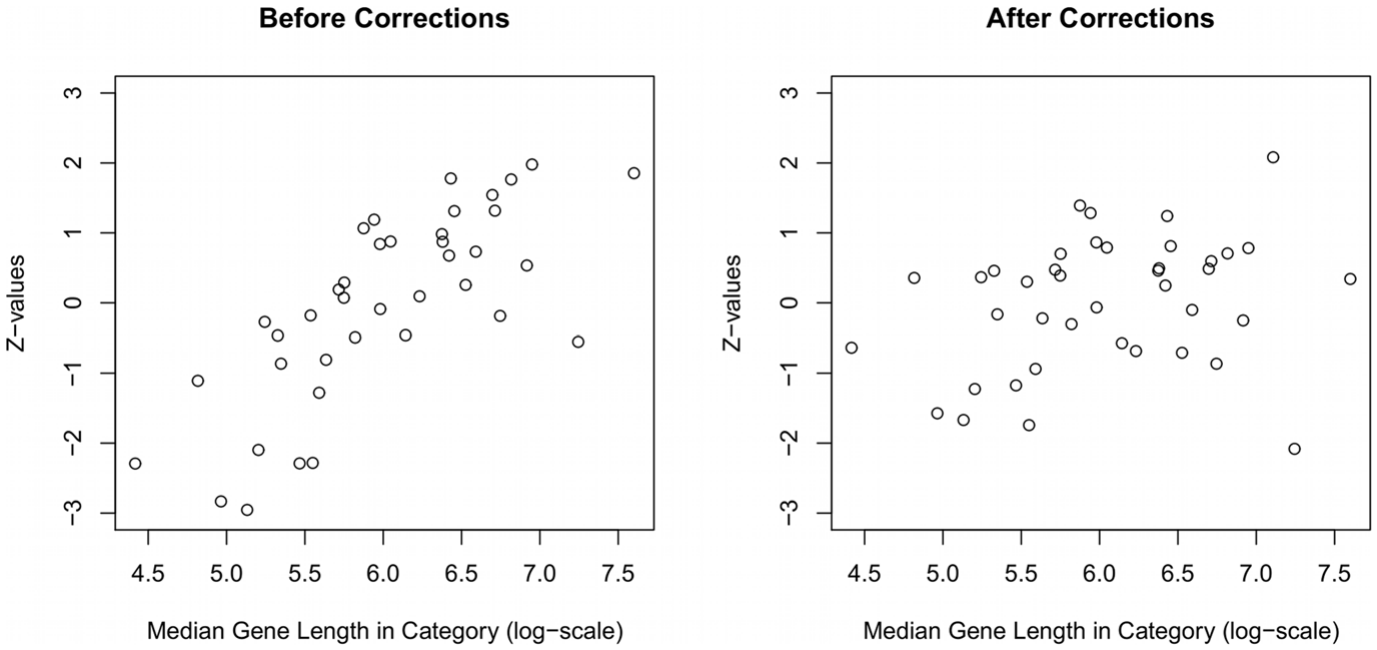
Scatter plots of the enrichment test statistic value against median gene length in category (log scale) before and after length bias corrections. Before length bias corrections, the enrichment test statistic value tends to increase with median gene length in category (left panel). After length bias corrections using GOglm, the trend is no longer visible (right panel).

In the absence of any length bias corrections, the simple logistic regression method identified a higher than expected number of categories with small 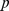-values (false positives; Figure 5). In contrast, with length bias corrections, both the GOglm and GOseq methods identified the correct proportions of small 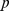-values as expected under this simulation. A further examination comparing the scatter plots of enrichment test statistic values versus median gene lengths across gene categories provided additional support that the correction methods addressed the problem of length bias (Figure 6). These scatter plots clearly revealed that the simple logistic method in the absence of any length bias corrections favored categories with longer genes whereas the GOglm method correctly adjusted for the length bias.

#### Simulation II

We use a second simulation to highlight an important difference between GOglm and GOseq. We also compared the performance of using different measures of DE test significance or different parameterizations of gene length in GOglm.

Here, we simulated six categories known to be enriched with equal proportions (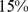) of DE genes (versus on average 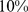 of DE genes in the other 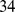 categories). However, we varied the degree to which the genes were differentially expressed: the DE genes in category one has the highest fold change whereas those in category six had the lowest fold change, but were still DE.

We compared the performance of the same three enrichment analysis approaches as in the previous example. However, with the GOglm method, we tested different measures of DE significance: untransformed DE test 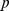-value, log fold change, log-transformed DE test 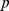-value, 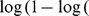
DE test 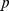-value)), and dichotomized DE 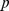-values (cut-off used was 0.05). We also examined the use of either lengths or log-transformed lengths as the covariate. We repeated the simulation 10 times and summarized the average ranks given to the six known enriched categories by the different approaches and parameterizations (Table 4). Based on the simulation results, we make the following conclusions:

1. The GOglm method with 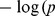-values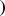 and log-transformed lengths (column 5) as covariates yielded the best performance: the known enriched categories were ranked highly and the ranking order reflected the degree of DE. When untransformed lengths were used with 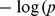-values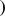 (column 6), the performance was very similar, but the average ranks given to the top two enriched categories were closer, indicating these two categories were less distinguishable under this setting.
2. The GOglm method with log fold change and log-transformed lengths (column 4) as covariates also performed well. Note that conditional on the mean level (which is proportional to gene length under this simulation setting), the 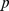-value is a monotone function of the log fold change. We should also be aware that fold changes are not available in regression settings (e.g., when investigating the dependence of expression level on a continuous covariate).
3. The GOglm method using dichotomized 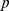-values perform very similar to the GOseq method (columns 9 and 10). Both methods ranked the known enriched categories highly, but the ranking orders did not reflect the degree of DE. This is as expected, since using dichotomized 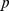-values retains only information on whether a gene is DE, not on how much the gene is DE. This can be viewed as a feature, rather than a drawback, of using dichotomized 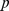-values, since sometimes it might be of interest to identify categories with a large number of DE genes rather than categories with a few extremely DE genes. Nevertheless, users should be aware of this interesting difference between using continuous 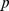-values and using dichotomized 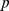-values.
4. Using the 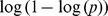 transformation (columns 7 and 8) ranked the top 3 enriched categories slightly lower than using the 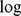-transformed 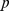-values (columns 5 and 6). In our real data examples, we used the 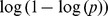 transformation to down weigh very extreme 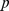-values. This became unnecessary for the simulated datasets, since we did not simulate very large fold changes. Again, we view this as a feature rather than a drawback. In practice, we recommend the initial use of scatter plots to help determine which transformations of 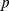-values and gene lengths are most suitable. One strength of the logistic regression method is that it allows different choices.
5. When length bias was not corrected (column 2), the logistic regression method still ranked the three enriched categories with the highest degree of DE highly, but the average ranks given to categories 4 and 5 were lower. This indicated that some non-enriched categories sometimes received higher rankings. As we now understand, this can happen due to length bias.
6. Use of untransformed 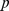-values (column 3) gave very poor performance. The degree of DE significance was not reflected well on the original scale of untransformed 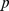-values.

**Table 4:** Average ranks of the six known enriched categories by different enrichment tests (over 10 simulations).

	Uncorrected	GOglm							GOseq
Significance^1^	−log(*p*)	−*p*	log_2_ FC	−log(*p*)	−log(*p*)	d-log	d-log	0/1	0/1
Length^2^	none	log(*l*)	log(*l*)	log(*l*)	*l*	log(*l*)	*l*	log(*l*)	s(*l*)
1	2.2	9.0	1.8	1.7	1.8	2.1	2.2	3.9	3.6
2	2.8	7.0	2.4	2.0	1.9	2.5	2.4	2.0	2.1
3	2.9	7.5	2.7	2.6	2.6	2.9	2.9	2.5	2.3
4	6.5	7.0	4.1	4.1	4.1	4.1	4.2	4.0	3.9
5	6.4	6.4	5.4	5.5	5.4	5.2	5.2	5.4	4.8
6	14.2	14.0	12.1	12.3	12.6	12.1	12.0	12.7	13.4

1 Significance: measures of DE significance. The measures used include minus untransformed DE test 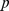-value, 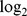 fold change, minus log-transformed DE test 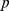-value, 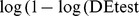
p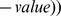 (d-log in the table header), and dichotomized DE 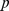-values (cut-off used was 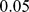).

2 Length: whether to use log-transformed lengths [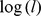] or original lengths (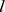); a smooth function 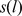 of length is used by GOseq for length corrections (the so-called probability weighting function, PWF).

## Discussion

The GOglm method discussed in this paper provides a simple and effective tool for GO enrichment analysis with simultaneous length bias corrections. The use of continuous measures of DE avoids the subjective specification of 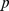-value thresholds that is inevitable for any contingency-table-based approaches. The GOglm is also applicable to local enrichment analyses which account for the parent-child relationship of the GO structure.

GO facilitates comprehensive and systematic functional explorations based on currently known biological knowledge. However, the complexity inherent in the ontology structure introduces statistical challenges from several different sources. Here we list a few issues that warrant deeper consideration beyond the scope of this article. Researchers should be aware of the potential influences these issues may have on inference before embracing any statistical tools available for enrichment analysis.

### Filtering Procedures

Not all genes under study had GO annotations available, and in this paper we restricted attention to study genes annotated with at least one GO term. In a GO enrichment analysis, researchers in general aim to determine which categories are more enriched relative to others in a database of all possible GO terms, so the selection is competitive in nature among the GO categories. Because genes without annotations tend to have relatively low expression and small variability across conditions, researchers may prefer to exclude them for limited discriminatory power and focus instead on the more studied genes. In the prostate cancer dataset, 10102 genes were without annotations, among which only 4.6% were called DE. In contrast, 16.9% of the 12592 genes having annotations were called DE.

Alternatively, researchers may choose to retain those genes without annotations in the study by assuming that they belong to a single “pseudo-category” and treating them as background genes. We note that analyses by GOseq in [10] and the goseq Bioconductor package did not exclude genes without annotations, so the final category list was slightly different than the list produced in this study.

### Challenges in GO Enrichment Analysis

#### Multiple testing correction

Because of the graph structure of GO terms, enrichment tests for different GO categories can be correlated, and therefore multiple testing corrections in GO analyses cannot be simply addressed by calculating ordinary false discovery rates (FDR) [21]. In our analysis, we did not adjust for multiplicity partially because we were more interested in relative ranks of categories instead of absolute magnitude of gene set 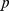-values.

Commonly used multiple testing corrections include the Holm-Bonferroni's correction [22], Benjamini-Hochberg's FDR [23], resampling-based 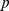-value adjustment [24], bootstrap and Monte-Carlo simulation approaches. Khatri *et al.* gave an overview of these corrections with an emphasis on their performances under different total numbers of functional categories and dependency levels [4]. Graph-structured tests have been discussed in [21] where the authors proposed a “focus level” method for DAG, and a hierarchical approach suitable for tree structures is proposed in [25]. Both methods are implemented in the Bioconductor package globaltest [26]–[28]. Han *et al.* also discussed false discovery control when test statistics are correlated [29]. Research on multiple corrections suitable for complex structures like GO is still in high demand.

#### Other sources of bias

In addition to length bias corrections, we can also implement GOglm in other situations such as corrections for total read counts of RNA-Seq data or corrections for intensity levels in microarray [10] in a similar manner. To obtain more accurate gene expression levels, Zheng *et al.* proposed in a recent paper a generalized-additive-model-based approach for simultaneous bias corrections from different sources, including gene lengths, GC content and dinucleotide frequencies [30]. We believe that such bias corrections performed in regression settings are promising, though the choice of appropriate regression tools depends on the underlying problem of interest.

#### Annotation quality

Even if the aforementioned statistical problems are properly addressed, the issue of annotation quality still exists as a non-statistical problem. Rhee *et al.* reported 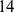 types of evidence supporting the association of GO identifiers to gene identifiers, with different levels of experimental validation [12]. Less than 5% of all annotations have been manually checked–which is considered as a reliable source of information. Over 95% of annotations, however, are indirectly derived (i.e. inferred from electronic annotation), leading to higher inaccuracy than those manually curated annotations (see Table 1 of [12]). Therefore, more efforts from biologists are needed to improve the annotation quality.

### Fundamental Assumption

One fundamental assumption underlying length bias corrections is that there is no biological cause for longer genes to be more differentially expressed, on average, than shorter genes. This assumption is not statistically verifiable, but both [9] and [10] cited evidence from microarray studies to support this assumption. If there is a biological cause that violates this assumption, then its effect will not be fully detected in GO enrichment analysis if length bias is corrected.

### Conclusion

We discussed a simple and effective method for length bias corrections in the GO enrichment analysis using logistic regression. We validated its effectiveness by analyzing real and simulated RNA-Seq datasets. We also compared its performance with alternative enrichment methods (e.g. GOseq) and examined the difference between the two approaches via simulations. Explicitly modeling the gene length as a covariate in the logistic regression framework helps to reduce length bias and enables flexible implementations and straightforward interpretations. The use of continuous measures of DE avoids the subjective specification of 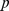-value thresholds. The method is flexible and applicable to local enrichment analyses which account for the parent-child relationship of the GO structure. making it a promising tool for enrichment analyses under different scenarios.

## Materials and Methods

### Preprocessing of the Prostate Cancer Dataset

The prostate cancer data [18] consist of seven samples: three from mock treated prostate cancer cells and four from treated cancer cells. The data originally consisted of 49605 genes as annotated using Ensembl gene ID (ensGene) and NCBI Build 36.3 (hg18). In order to directly compare GOglm to GOseq, we did not attempt to remap the reads to the genome. We fetched gene lengths and mapped gene identifiers to GO terms from available annotation Bioconductor packages. For example, we used the org.Hs.egGO2ALLEGS R object in the Bioconductor annotation package org.Hs.eg.db to obtain mappings between a given GO category and all its annotated Entrez Gene identifiers [31]. Gene length information is also accessible from the Ensembl Project online. Descriptions of data preparations can be found in the additional file of [10].

The 49506 genes under study were first filtered by excluding genes with no fold changes. Among the remaining 22743 genes, 12592 genes had GO annotations for a total of 13956 unique GO categories. In our GO enrichment analyses, we excluded 9162 categories (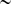
69.8%) with fewer than 10 annotated genes from the final enrichment ranking list. Statistical and biological considerations for gene subsetting were mentioned in the Results and Discussion sections, respectively.

### Preprocessing of the Arabidopsis Dataset

The org.At.tairGO2ALLTAIRS R object in the Bioconductor annotation package org.At.tair.db provides mappings between a GO category and all its annotated TAIR identifiers [32]. We subsetted our dataset from a total of 6916 GO terms available in the database.

Testing a GO term's relative enrichment requires knowledge of this term's direct parental term(s), and this information is available from the GO.db Bioconductor package [33]. We found in our Arabidopsis dataset 3993 BP, 601 CC and 2322 MF terms each having at least one annotated gene.

We excluded 2039 genes that had zero read counts in all samples, and discarded an additional 909 genes with zero fold change. We further excluded 2504 genes without annotations, so that the original 26222 genes were subsetted into 20770 genes associated with 6916 unique categories. Median transcript lengths for all genes were available. In this example, we excluded categories with fewer than 4 genes (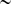
50%) and focused on 3851 categories for enrichment analysis.

### Assessment of Differential Gene Expression

For ease of comparison with published results, in analyzing the prostate cancer data [18], we used edgeR [1] with a common dispersion estimate to obtain DE test 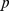-values. For the Arabidopsis dataset, we used NBPSeq [3] to obtain DE test 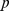-values. EdgeR and NBPSeq are both based on negative binomial models for RNA-Seq read frequencies. The negative binomial model captures potential extra-Poisson variation in RNA-Seq read frequencies between independent biological samples using a dispersion parameter. Other methods based on negative binomial model include the tagwise or trend options in edgeR, or the DESeq approach discussed in [2]. All of these methods use the same exact NB test [34] for assessing DE, but differ in how they estimate the dispersion parameter as a function of the mean frequency.

### Simulation I

We simulated a 10426 gene dataset. The genes were binned into 40 non-overlapping categories with the number of genes ranging from 101 to 1252. To keep the simulation simple and focused on the issue of length bias, we simulated non-overlapping categories to avoid correlated test statistics. In addition, moderate sizes of simulated categories provide adequate statistical power in the enrichment analysis. Gene lengths (on the log scale) were simulated according to a normal distribution with mean 6 and standard deviation 0.7 (simulated lengths ranged from 20 to 5867 base pairs). We assigned genes with the shortest lengths to the first category, genes with the second shortest lengths to the second category, and so on. The last category therefore contained the longest genes. After assigning genes to categories, we added additional noise to the log gene length according a normal distribution with mean 0 and standard deviation 0.2 so that there was overlap between gene length distributions in different gene categories. This additional step was useful to avoid potential convergence issues in the logistic regression.

We simulated RNA-Seq read counts according to negative binomial distributions for 12 biological samples divided into two groups, each of size 6. We specified the expected values 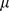 of read counts to be proportional to simulated gene lengths and the dispersion parameter 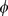 as a function of the mean 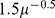. This dispersion model mimicked the one estimated for the Arabidopsis data in [19]. We randomly designated 20% of all genes as DE. The proportion of DE genes in each category varied from 14% and 26% due to chance variations, but was independent of the gene length distribution. For the DE genes, the mean values of read counts in one of the groups (randomly decided) was less than that in the other group, and the expected log fold change (base 2) between the two groups was 0.5. We performed DE tests using NBPSeq. The resulting 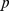-values were transformed into a significance measure using 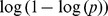 and used as one covariate in the logistic regression for testing category enrichment.

### Simulation II

We simulated 40 gene categories with the same category sizes and gene length distributions as in the first simulation. The baseline mean levels of simulated RNA-Seq reads were again proportional to simulated gene lengths. However, this time we simulated six categories to be enriched with DE genes. In each of these six categories, there were 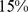 of DE genes. The remaining 34 categories had 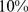 of DE genes (randomly simulated among all genes, so the actual number of DE genes in each of these 34 categories followed a binomial distribution with probability 0.1). We assigned a constant 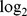 fold change of 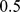 to DE genes in the non-enriched categories, but the 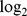 fold changes of DE genes in the six enriched categories ranged from 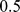 to 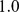 at an increment of 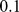, resulting in varying degrees of DE among these categories.

### Software Information

The R codes implementing GOglm are available at the first author's website: http://people.oregonstate.edu/ 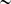
mig/Site/Research.html. GOglm conforms to the definition of Open Source as defined by the Open Source Initiative. We have licensed GOglm under the GNU General Public License.

## Supporting Information

Table S1 **Complete GO ranking list by GOglm.**
(XLS)

Table S2 **Complete GO ranking list by Ontologizer2 (PCU).**
(XLS)
